# Tape‐Stripped Proteomics: A Comparative Analysis of Different Sample Collection Approaches

**DOI:** 10.1002/pmic.70177

**Published:** 2026-09-15

**Authors:** Ali Azimi, Lauren Faul, Petrina Chand, Pablo Fernandez‐Penas

**Affiliations:** ^1^ Department of Dermatology Westmead Hospital University of Sydney Sydney New South Wales Australia; ^2^ Centre For Cancer Research The Westmead Institute For Medical Research Sydney New South Wales Australia; ^3^ Department of Dermatolgoy, Westmead Hospital Westmead Clinical School Faculty of Medicine and Health The University of Sydney Sydney New South Wales Australia

**Keywords:** DIA‐MS, stratum corneum, tape‐stripping

## Abstract

Tape‐stripping of the stratum corneum is a promising non‐invasive approach for proteomic analysis of skin lesions, but the effects of sampling design and disc pressure on proteome readout remain poorly defined. Here, we assessed how inclusion of surrounding normal‐looking skin and variation in adhesive disc pressure influence protein detection from small melanocytic lesions. D‐squame tape strips were collected from six participants, including three moles and one matched normal skin site per participant. For lesion 1 (L1), discs were applied using light finger pressure. For lesion 2 (L2), the mole area was marked on the disc and separated from the surrounding skin, which was analysed separately (L2S). For lesion 3 (L3), increased pressure was applied using the upper barrel tip of a marker before excising the lesion‐matched area. Samples were analysed by DIA‐MS with DIA‐NN processing. In total, 2882 protein groups were identified, including 1719 shared across all groups. L3 yielded the highest number of unique proteins (77) and significantly altered proteins versus normal skin (142), compared with L2 (54 unique; 52 altered), L1 (30 unique; 4 altered), L2S (16 unique; 3 altered), and normal skin (7 unique). These findings show that precise lesion‐matched disc trimming and increased application pressure substantially improve tape‐strip proteomic depth and lesion‐specific signal detection. Multivariate analysis and supervised classification further separated lesional samples—most clearly the pressure‐assisted L3—from other collection methods, supporting these observations, although the small cohort warrants cautious interpretation.

The analysis of human stratum corneum samples collected by the application of adhesive discs (tape‐stripping) is increasingly reported in skin research. These non‐invasively collected samples have been successfully used to detect genes, transcripts and protein signatures related to different skin diseases, including skin cancers and inflammatory skin conditions [1, 2, 3, 4, 5, 6]. Most benign and early malignant skin lesions are small and vary in shape and size, making them difficult to match with the available standard adhesive disc sizes. Therefore, the collection of surrounding normal‐looking skin cannot be avoided. Moreover, the application of varying levels of pressure on the adhesive disc can impact the protein content and identification of protein signatures, leading to reduced reproducibility.

Therefore, the current study evaluated the effect of sampling clinically normal‐looking skin samples surrounding moles and the application of different amounts of pressure on the adhesive discs on the measured proteome from stratum corneum samples. In total, tape‐strip samples were collected from the forearm of six participants, comprising three different moles and one matched normal‐skin site per participant, using D‐squame adhesive discs (D104, D‐squame, Clinical and Derm, Dallas TX, USA) as described before [3]. For lesion one (L1; mole), light pressure using a finger was applied to five adhesive discs sequentially placed on the lesion and peeled off. The step was repeated for lesion two (second mole), however, the adhesive disc area covered by the mole was marked with a marker (Sharpie, Fine point, Tennessee, USA), followed by separating the marked area (L2) from the surrounding area (L2S) with surgical scissors. For lesion three (third mole; L3), the amount of pressure applied to the lesion was increased using the upper barrel tip of a permanent marker (Sharpie). The area within the marked area was cut and retained for downstream analysis. Sample four (normal skin; NS) included matched normal skin collected from the patient's forearm, with light pressure using a finger. An overview of the sample collection and downstream analysis is presented in Figure 1. All participants provided written informed consent (Western Sydney Local Health District ethics approval #HREC/17/WMEAD/555).

**FIGURE 1:**
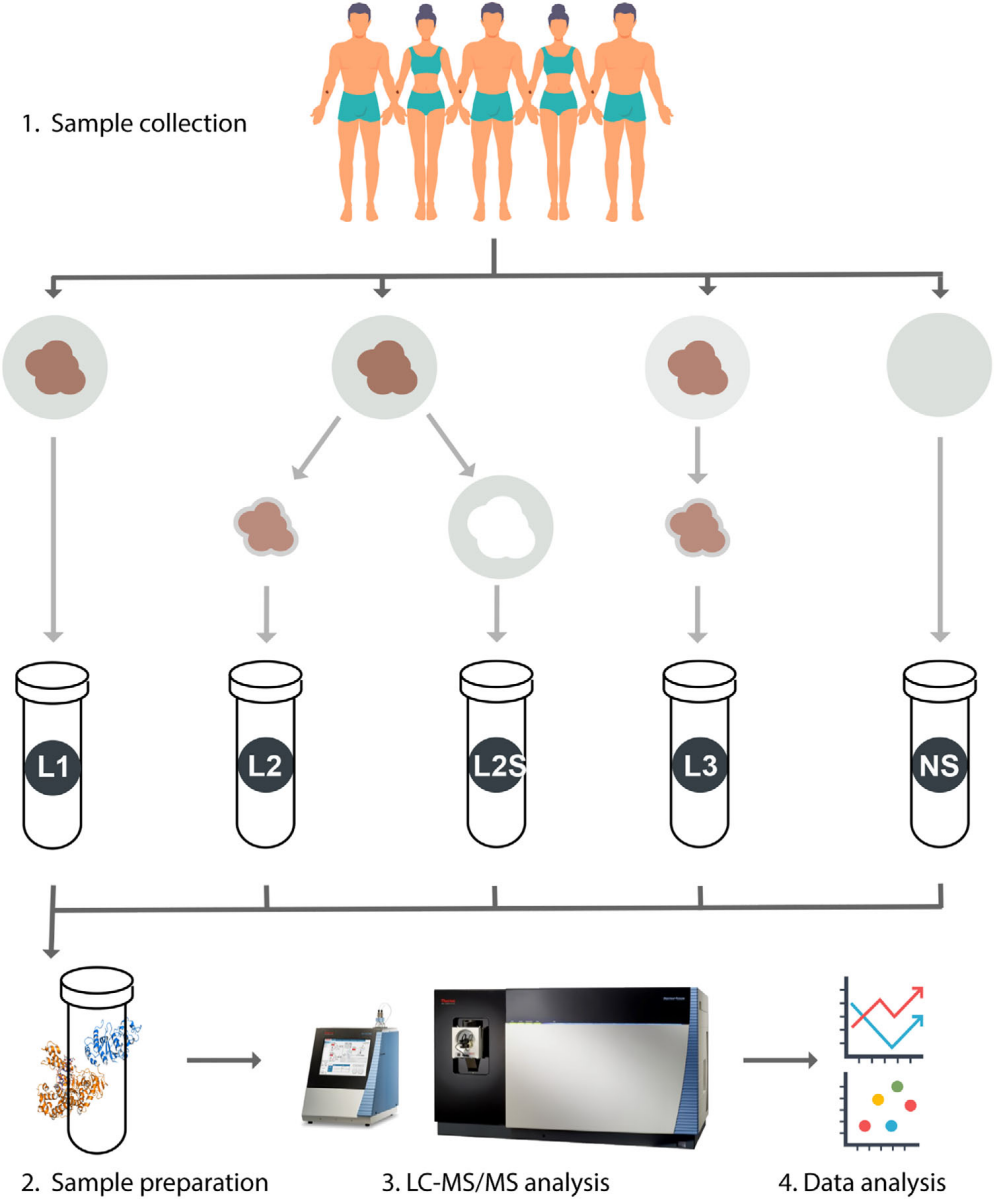
An overview of the sample collection, sample preparation and downstream proteomic analysis. L1: Mole collected using finger pressure on the adhesive disc, uncut; L2: Mole collected using the same pressure as L1, but with the adhesive disc cut around the lesion; L2S: Normal‐looking skin surrounding L2; L3: Mole collected using pressure from the upper barrel tip of a permanent marker, with the adhesive disc cut around the lesion; NS: normal skin collected using the same pressure as L1.

Protein contents from the discs were dissociated, cleaned and digested by trypsin in S‐trap columns (Protifi, Fairport NY, USA) as described before [7]. Final quantitation was determined using the Qubit protein assay (Thermo Fisher Scientific, North Ryde, NSW, Australia). The protein digests (0.6 ug each) were separated using a nanoflow HPLC system (Ultimate 3000, Dionex, Idstein, Germany) coupled to an Orbitrap Fusion Lumos Tribrid mass spectrometer (Thermo Fisher Scientific) over a 90 min gradient using a data‐independent acquisition (DIA) strategy as described earlier [7]. The proteomic data were processed for protein identification and label‐free quantitation using DIA‐NN (Version 1.9) [8] with default parameters, and the human fasta file (UniProt, 10/01/2025) was used as the reference proteome. A library‐free search strategy was employed, whereby an in silico spectral library was predicted directly from the UniProt human FASTA using the DIA‐NN deep‐learning predictor. Cross‐run normalisation was applied within DIA‐NN (RT‐dependent normalisation), and equal peptide inputs were loaded per sample so that the Qubit assay served to standardise loading rather than for downstream scaling. Proteins with a maximum of 1% FDR (applied at both the precursor and protein‐group level) were exported into a Microsoft Excel file for downstream analysis. Following log_2_‐transformation, the data were subjected to differential abundance (LIMMA) analysis in WebMev [9].

In total, 2882 unique protein groups were detected, of which 1719 proteins (59.65%) were common between all sample groups (Figure 2a). L3 lesions collected using the highest pressure identified 77 unique protein groups, followed by L2 (54) and L1 (30), indicating improvements in total protein identification when extra pressure is applied to the adhesive discs. L2S (16), the normal‐looking skin surrounding the mole, and NS (7) identified the lowest number of unique protein groups, indicating that intact skin yields fewer proteins due to its unaltered state.

**FIGURE 2:**
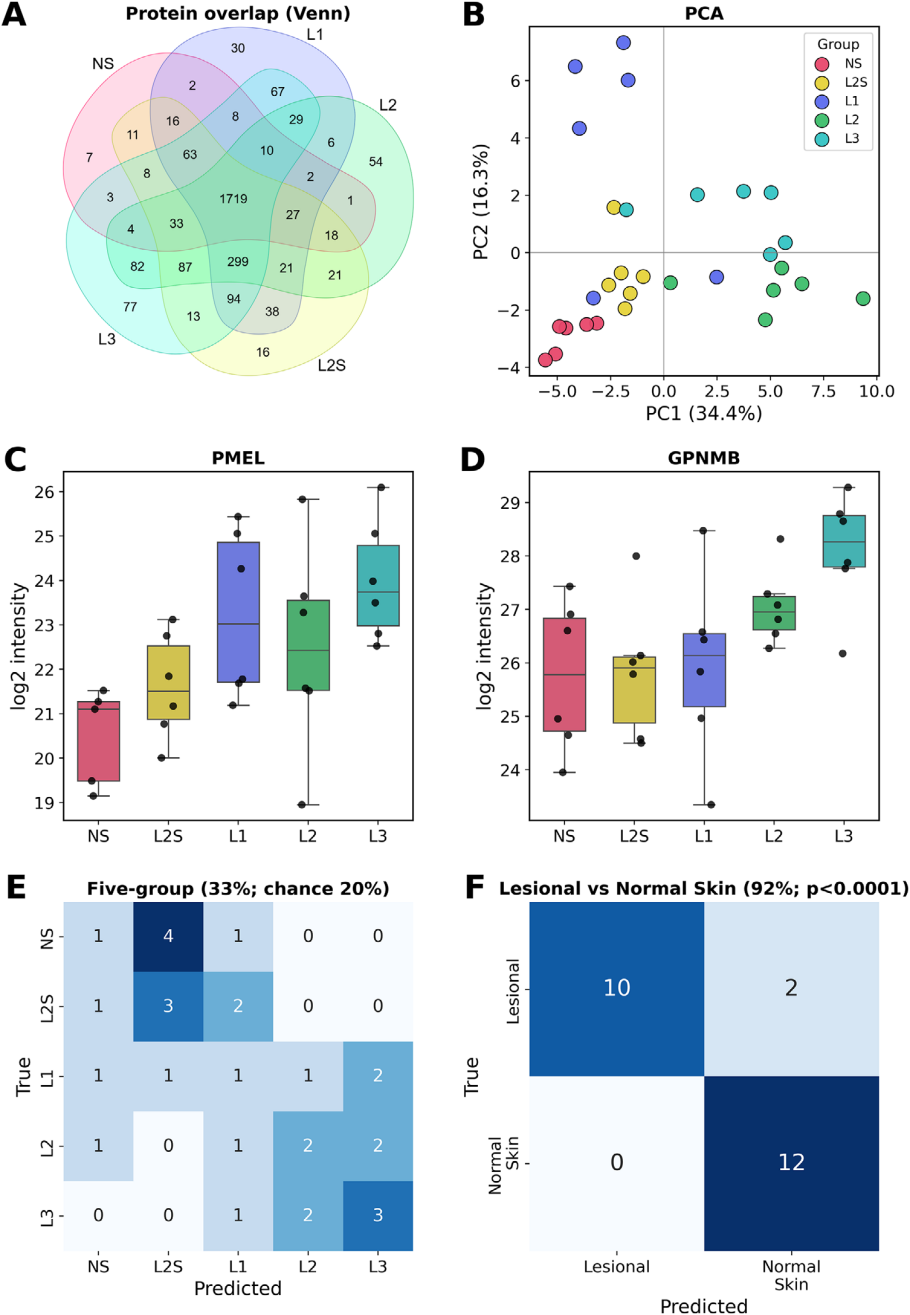
Multivariate and lesion‐specific analysis of tape‐strip proteomes. (A) Venn diagram of protein groups identified across the five groups (1719 shared). (B) PCA of the 50 most group‐discriminating protein groups (ranked by ANOVA F‐statistic); PC1 separates normal skin (NS) and normal skin surrounding the lesions (L2S) from the lesional samples (L2 and L3), with L1 intermediate. (C,D) Abundance (log2 intensity) of the melanocyte‐derived proteins PMEL (C) and GPNMB (D) by group, both increasing toward the pressure‐assisted, lesion‐matched preparation (L3). (E,F) Leave‐one‐out cross‐validated classification (feature selection within each fold): (E) five‐group confusion matrix (accuracy 33%; chance 20%), with errors concentrated between biologically similar groups (NS and L2S; L2 and L3); (F) lesional (L2 + L3) versus normal skin (NS + L2S), 92% accuracy (*p* < 0.0001). Rows = true group, columns = predicted, cells = sample counts.

Using the normal skin sample as a control, differential abundance analysis using LIMMA identified four proteins significantly changed in L1, 52 in L2, three in L2S, and 142 in L3 (adj. *p* value<0.05; log_2_ of absolute fold change >1) (Table 1 and Table S1). Two proteins changed in L1 and two proteins changed in L2S were also changed in either L2 or L3 lesions. Together, the results indicate that increasing pressure on the adhesive disc, combined with cutting around the lesion, improves the likelihood of detecting more significantly altered proteins in moles. Additionally, the results suggest that the normal‐looking skin surrounding the lesion differs from healthy skin. This could either be due to changes in the skin cells or a potential collection of mole cells, given their proximity to the lesion. Of the top‐25 proteins changed in L2 (ranked by adj. *p* value), 18 of them were also found to have changed in L3 samples (same direction of change) (Table 1 and Table S1), implying that while both approaches detect changes in similar proteins, the number of changes identified by L3 is significantly greater, suggesting it to be a better approach for in‐depth proteomic analysis of moles.

**TABLE 1:** The list of protein groups changed in L1, L2, L2S and L3 versus normal skin (adj. *p* value < 0.05; log_2_ of absolute fold change > 1).

Proteins	log2 fold change	Adj. *p* value
*The list of protein groups changed in L1 versus normal skin*		
^a^, ^b^PSMD14	3.06	7.54E‐03
^a^ATP6V1H	3.02	7.54E‐03
PRH1	−3.33	7.54E‐03
HERC1	3.06	1.37E‐02
		
*The list of top 20 protein groups changed in L2 versus normal skin*		
^a^DEFA1; DEFA3	−3.93	2.21E‐18
IGHV3‐30; IGHV3‐35	−3.84	2.02E‐17
^a^PLA2G2A	−3.82	5.10E‐17
^a^CD59	−3.29	6.77E‐03
SPRR2F	−3.18	3.21E‐03
CEACAM19	−3.11	5.73E‐03
RPTN	−2.66	3.79E‐02
IGHG3	−2.61	3.79E‐02
^a^SRSF1	2.58	3.79E‐02
^a^RBM39	2.59	3.79E‐02
CPSF7	2.59	3.79E‐02
^a^U2AF1	2.6	3.79E‐02
CPSF6	2.63	3.79E‐02
LUC7L2	2.63	3.79E‐02
^a^RPL39; RPL39P5	2.64	3.79E‐02
^a^TCP1	3.11	3.83E‐03
FKBP2	3.16	3.17E‐03
SRSF2	3.23	8.56E‐03
SRSF3	3.29	2.72E‐02
^a^RPL23A	3.30	3.17E‐03
		
*The list of protein groups changed in L2S versus normal skin*		
CHIC2	3.14	4.32E‐03
^a^DPM1	3.05	5.58E‐03
^b^SRSF2	3.09	7.63E‐03
		
*The list of top 20 protein groups changed in L3 versus normal skin*		
^b^PLA2G2A	−3.83	1.81E‐12
^b^DEFA1; DEFA3	−3.28	2.64E‐03
PRPF19	3.12	3.15E‐03
^b^ARCN1	3.13	3.15E‐03
^b^TARS1	3.14	3.15E‐03
PSMD14	3.14	3.20E‐03
EIF3C	3.15	3.15E‐03
PACSIN2	3.15	3.20E‐03
CCT7	3.16	2.64E‐03
RPS23	3.16	2.64E‐03
EIF3D	3.18	3.20E‐03
RPL27A	3.20	2.64E‐03
^b^SRSF1	3.20	3.20E‐03
^b^RPL32	3.22	3.39E‐03
RPL23A	3.26	4.03E‐03
RPS5	3.28	4.41E‐03
RPS26	3.34	3.15E‐03
^b^RPL39; RPL39P5	3.63	1.67E‐10
ETF1	3.66	1.81E‐12
DPM1	3.66	2.17E‐12

^a^ Also changed in L3 versus normal skin.

^b^ Also changed in L2 versus normal skin.

To provide a global multivariate overview of the sampling approaches, principal component analysis (PCA) was performed on the most group‐discriminating protein groups (*n* = 50) selected by their one‐way ANOVA F‐statistic across the five groups. Log2 intensities were used with missing values set to the global minimum, and the selected protein groups were standardised (z‐scored) prior to PCA. Samples separated along the principal components according to tissue type, with NS and normal skin surrounding the lesions (L2S) clustering apart from the lesional samples (L2 and L3), while L1 occupied an intermediate position, consistent with its mixed lesion‐plus‐surrounding composition (Figure 2b). Consistent with enrichment of lesion‐specific signal, the melanocyte‐derived proteins PMEL and GPNMB increased progressively from NS to the pressure‐assisted, lesion‐matched preparation (L3) (Figure 2c,d). Finally, to test whether the sampling groups could be distinguished from their proteomes, samples were classified under leave‐one‐out cross‐validation, with feature selection performed within each training fold to prevent information leakage. Using a random‐forest classifier, classification across all five groups was modest (33% accuracy; chance 20%), with misclassifications occurring predominantly between biologically similar groups (NS vs. L2S and L2 vs. L3); by contrast, binary classification of lesional (L2 + L3) versus normal skin (NS + L2S), using elastic‐net‐regularised logistic regression and sparse partial least squares discriminant analysis (sPLS‐DA), achieved 92% cross‐validated accuracy (22 of 24 samples; exact binomial p < 0.0001; Figure 2e,f). Together, these analyses reinforce the idea that increased pressure and lesion‐matched trimming enhance lesion‐specific proteomic signal, although the small cohort warrants cautious interpretation.

Altogether, findings from the current study show that for an in‐depth proteomic analysis of skin lesions using tape‐stripping, it is important that the size of the adhesive collection disc is adjusted to match the size of the lesion being sampled, and that sufficient pressure is applied to the disc. The use of pressure devices, such as those provided by Clinical and Derm (Dallas, TX, USA), while useful for small‐scale studies, may not be practical when samples are to be collected by patients themselves at home due to the increased cost. Therefore, the use of alternative, basic tools, such as the upper barrel tip of a marker, could offer a cheaper and viable option.

This study has limitations that should be considered when interpreting the results. First, the cohort was small (six participants), which limits statistical power and generalisability; the present findings should therefore be regarded as exploratory and require confirmation in larger, independent cohorts. Second, although the barrel‐tip approach reproducibly increased the pressure applied to the disc, the absolute force was not instrument‐controlled and may vary between collections; incorporating a calibrated pressure device in future work would allow this variable to be standardised and quantified.

In conclusion, the findings from this exploratory study will inform the efficient and thoughtful design of tape‐strip sample collection for biomarker analysis. This will be particularly beneficial for research groups with multiple collection sites or those focused on the collection of tape strips by individual patients in the comfort of their own homes.

## Conflicts of Interest

The authorsdeclare no conflicts of interest.

## Supporting information




**Supporting File**: pmic70177‐sup‐0001‐TableS1.docx

## Data Availability

The mass spectrometry proteomics data have been deposited in the jPOSTrepo (Japan ProteOme STandard Repository) [10], an international standard data repository for proteomes. The accession numbers are PXD057504 for ProteomeXchange and JPST003450 for jPOST.
