# Supplementary material for: Tape‐Stripped Proteomics: A Comparative Analysis of Different Sample Collection Approaches
Source: Proteomics. 2026 Sep 15;26(11):35–9. doi: 10.1002/pmic.70177 (PMC13721137; doi:10.1002/pmic.70177)
Supplement: Supplementary file 1 [file PMIC-26--s001.docx]

**Tape-Stripped Proteomics: A Comparative Analysis of Different Sample Collection Approaches**

**Supplementary Table**

Supplementary Table 1: Complete list of proteins changed in L1, L2, L2S and L3 versus normal skin (adj. p-value<0.05; log_2_ of absolute fold change >1)

| **Proteins** | **log2 Fold Change** | **Adj. p-value** |
| --- | --- | --- |
| *The list of proteins changed in L1 versus normal skin* | | |
| *PSMD14 | 3.06 | 7.54E-03 |
| *ATP6V1H | 3.02 | 7.54E-03 |
| PRH1 | -3.33 | 7.54E-03 |
| HERC1 | 3.06 | 1.37E-02 |
|  |  |  |
| *The list of top 20 proteins changed in L2 versus normal skin* | | |
| *DEFA1; DEFA3 | -3.93 | 2.21E-18 |
| IGHV3-30; IGHV3-35 | -3.84 | 2.02E-17 |
| *PLA2G2A | -3.82 | 5.10E-17 |
| FKBP2 | 3.16 | 3.17E-03 |
| *RPL23A | 3.3 | 3.17E-03 |
| SPRR2F | -3.18 | 3.21E-03 |
| *TCP1 | 3.11 | 3.83E-03 |
| CEACAM19 | -3.11 | 5.73E-03 |
| *CD59 | -3.29 | 6.77E-03 |
| *AK2 | 2.48 | 3.79E-02 |
| *ARCN1 | 2.46 | 3.79E-02 |
| ABCE1 | -2.59 | 3.79E-02 |
| *DHX15 | 2.57 | 3.79E-02 |
| *ELAVL1 | 2.54 | 3.79E-02 |
| *HDLBP | 2.5 | 3.79E-02 |
| *HP1BP3 | 2.58 | 3.79E-02 |
| *SRSF1 | 2.58 | 3.79E-02 |
| *TARS1 | 2.48 | 3.79E-02 |
| ATP5PO | 2.55 | 3.79E-02 |
| *RPL39; RPL39P5 | 2.64 | 3.79E-02 |
| *TOP1 | 2.57 | 3.79E-02 |
| *PCMT1 | 2.44 | 3.79E-02 |
| *PSPC1 | 2.57 | 3.79E-02 |
| *RBM39 | 2.59 | 3.79E-02 |
| *RPL32 | 2.56 | 3.79E-02 |
| SRSF2 | 3.23 | 8.56E-03 |
| SRSF3 | 3.29 | 2.72E-02 |
| CPSF6 | 2.63 | 3.79E-02 |
| CPSF7 | 2.59 | 3.79E-02 |
| *U2AF1 | 2.6 | 3.79E-02 |
| IGHG3 | -2.61 | 3.79E-02 |
| DCTN2 | 2.47 | 3.79E-02 |
| DDX46 | 2.53 | 3.79E-02 |
| LUC7L2 | 2.63 | 3.79E-02 |
| RPS28 | 2.53 | 3.79E-02 |
| RPTN | -2.66 | 3.79E-02 |
| SORBS2 | 2.57 | 3.79E-02 |
| U2AF2 | 2.57 | 3.79E-02 |
| *CCT8 | 2.56 | 3.84E-02 |
| RALY | 2.4 | 3.84E-02 |
| ATP5F1D | 2.47 | 3.90E-02 |
| CA1 | -2.51 | 3.90E-02 |
| *CCN3 | -2.49 | 3.90E-02 |
| CEACAM7 | -2.47 | 3.90E-02 |
| CRCT1 | -2.57 | 3.90E-02 |
| IGHV1-18 | -2.52 | 3.90E-02 |
| *PRB3 | -2.53 | 3.90E-02 |
| PSCA | -2.46 | 3.90E-02 |
| SDCBP | -2.39 | 3.90E-02 |
| SELENOI | -2.42 | 3.90E-02 |
| SUCLG2 | 2.42 | 3.90E-02 |
| UBE2N | 2.45 | 3.90E-02 |
|  |  |  |
| *The list of proteins changed in L2S versus normal skin* | | |
| CHIC2 | 3.14 | 4.32E-03 |
| *DPM1 | 3.05 | 5.58E-03 |
| ^SRSF2 | 3.09 | 7.63E-03 |
|  |  |  |
| *The list of top 20 proteins changed in L3 versus normal skin* | | |
| *PLA2G2A | -3.83 | 1.81E-12 |
| *DEFA1; DEFA3 | -3.28 | 2.64E-03 |
| *CD59 | -2.67 | 2.33E-02 |
| FMOD | -2.57 | 2.33E-02 |
| *PRB3 | -2.54 | 2.33E-02 |
| *CCN3 | -2.5 | 2.33E-02 |
| IGFBP7 | -2.5 | 2.33E-02 |
| FAM83G | 2.24 | 2.33E-02 |
| PLAA | 2.27 | 2.33E-02 |
| AK4 | 2.28 | 2.33E-02 |
| PIGO | 2.28 | 2.52E-02 |
| GATD1 | 2.32 | 2.33E-02 |
| DCTN5 | 2.32 | 2.33E-02 |
| PRKAR1A | 2.34 | 2.33E-02 |
| PTK6 | 2.34 | 2.33E-02 |
| TMEM41B | 2.34 | 2.49E-02 |
| LYPLAL1 | 2.34 | 2.33E-02 |
| HIBCH | 2.34 | 2.33E-02 |
| TSG101 | 2.35 | 2.33E-02 |
| NDUFA9 | 2.36 | 2.33E-02 |
| TM9SF1 | 2.36 | 2.50E-02 |
| NEMF | 2.36 | 2.47E-02 |
| DERL1 | 2.36 | 2.33E-02 |
| RUVBL2 | 2.37 | 2.33E-02 |
| STK24 | 2.37 | 2.33E-02 |
| DIP2B | 2.38 | 2.33E-02 |
| ARPC5L | 2.38 | 2.33E-02 |
| BPNT1 | 2.38 | 2.33E-02 |
| NMT1 | 2.38 | 2.33E-02 |
| EIF2A | 2.38 | 2.33E-02 |
| SEC63 | 2.39 | 2.33E-02 |
| NCKAP1 | 2.39 | 2.33E-02 |
| WDR26 | 2.39 | 2.33E-02 |
| RBM25 | 2.39 | 2.33E-02 |
| TMX4 | 2.39 | 2.45E-02 |
| *AK2 | 2.40 | 2.33E-02 |
| PGLS | 2.40 | 2.34E-02 |
| ATP6V1C2 | 2.40 | 2.47E-02 |
| TXNDC5 | 2.40 | 2.33E-02 |
| DCAF7 | 2.41 | 2.47E-02 |
| TPP2 | 2.41 | 2.33E-02 |
| LARS1 | 2.42 | 2.33E-02 |
| ETFB | 2.42 | 2.33E-02 |
| SEH1L | 2.42 | 2.33E-02 |
| MAP2 | 2.42 | 2.33E-02 |
| EIF4A3 | 2.42 | 2.33E-02 |
| SEC31A | 2.42 | 2.33E-02 |
| MAPK1 | 2.42 | 2.33E-02 |
| EIF5 | 2.42 | 2.33E-02 |
| HS2ST1 | 2.43 | 2.34E-02 |
| EIF3L | 2.43 | 2.33E-02 |
| *PCMT1 | 2.43 | 2.33E-02 |
| HIP1R | 2.43 | 2.33E-02 |
| RHOG | 2.43 | 2.33E-02 |
| AARS1 | 2.43 | 2.33E-02 |
| NDUFS1 | 2.44 | 2.33E-02 |
| ERAP1 | 2.44 | 2.47E-02 |
| IGKV1-33; IGKV1D-33 | 2.44 | 2.33E-02 |
| EIF1AX; EIF1AY | 2.44 | 2.33E-02 |
| SBDS | 2.44 | 2.33E-02 |
| NLRP10 | 2.44 | 2.47E-02 |
| EIF4E | 2.45 | 2.33E-02 |
| HMGCS1 | 2.45 | 2.33E-02 |
| HYOU1 | 2.45 | 2.33E-02 |
| SARS1 | 2.45 | 2.33E-02 |
| LIPA | 2.45 | 2.33E-02 |
| HNRNPUL1 | 2.45 | 2.33E-02 |
| GNA15 | 2.45 | 2.97E-02 |
| *HDLBP | 2.45 | 2.33E-02 |
| DDX6 | 2.46 | 2.33E-02 |
| CSDE1 | 2.46 | 2.33E-02 |
| *RBM39 | 2.47 | 2.33E-02 |
| EIF3A | 2.47 | 2.33E-02 |
| *U2AF1 | 2.47 | 2.33E-02 |
| *ELAVL1 | 2.47 | 2.33E-02 |
| *PSPC1 | 2.47 | 2.33E-02 |
| FDPS | 2.48 | 2.33E-02 |
| RCC2 | 2.48 | 2.55E-02 |
| *TOP1 | 2.48 | 2.33E-02 |
| VAPA | 2.48 | 2.33E-02 |
| USO1 | 2.48 | 2.33E-02 |
| *HP1BP3 | 2.49 | 2.33E-02 |
| COPG1 | 2.49 | 2.33E-02 |
| PSMD7 | 2.49 | 2.33E-02 |
| AKR1C2 | 2.50 | 2.33E-02 |
| CTNNA1 | 2.50 | 2.33E-02 |
| MAPK3 | 2.50 | 2.33E-02 |
| COPA | 2.50 | 2.42E-02 |
| LMO7 | 2.51 | 2.33E-02 |
| EPRS1 | 2.51 | 2.33E-02 |
| IGHG4 | 2.52 | 2.33E-02 |
| EEF1B2 | 2.53 | 2.33E-02 |
| YWHAE | 2.54 | 2.33E-02 |
| RPS8 | 2.54 | 2.71E-02 |
| PSMD2 | 2.54 | 2.33E-02 |
| GRHPR | 2.56 | 4.91E-02 |
| *CCT8 | 2.56 | 2.33E-02 |
| ACLY | 2.56 | 3.78E-02 |
| FN1 | 2.57 | 3.16E-02 |
| COPB1 | 2.59 | 2.33E-02 |
| CNOT1 | 2.75 | 5.91E-03 |
| PRPS1 | 2.83 | 6.70E-03 |
| GSDMC | 2.9 | 3.15E-03 |
| GOLGA7 | 2.97 | 2.94E-03 |
| PSMD6 | 2.99 | 2.70E-03 |
| ATIC | 3.00 | 2.94E-03 |
| TMEM79 | 3.00 | 3.20E-03 |
| PSMC5 | 3.00 | 2.94E-03 |
| CUL3 | 3.01 | 2.94E-03 |
| BZW2 | 3.01 | 2.94E-03 |
| RUVBL1 | 3.04 | 2.64E-03 |
| SAR1A | 3.05 | 2.64E-03 |
| DNM2 | 3.06 | 2.64E-03 |
| ENO2 | 3.09 | 2.70E-03 |
| PRPF8 | 3.09 | 2.64E-03 |
| VPS35 | 3.09 | 2.64E-03 |
| ATP6V1H | 3.10 | 3.15E-03 |
| *TCP1 | 3.10 | 2.64E-03 |
| RPL9 | 3.10 | 2.94E-03 |
| ASL | 3.11 | 3.15E-03 |
| *DHX15 | 3.11 | 3.15E-03 |
| PPP2R1A | 3.11 | 3.15E-03 |
| ADK | 3.11 | 3.15E-03 |
| RBBP4 | 3.11 | 3.15E-03 |
| PRPF19 | 3.12 | 3.15E-03 |
| *ARCN1 | 3.13 | 3.15E-03 |
| *TARS1 | 3.14 | 3.15E-03 |
| PSMD14 | 3.14 | 3.20E-03 |
| EIF3C | 3.15 | 3.15E-03 |
| PACSIN2 | 3.15 | 3.20E-03 |
| CCT7 | 3.16 | 2.64E-03 |
| RPS23 | 3.16 | 2.64E-03 |
| EIF3D | 3.18 | 3.20E-03 |
| RPL27A | 3.20 | 2.64E-03 |
| *SRSF1 | 3.20 | 3.20E-03 |
| *RPL32 | 3.22 | 3.39E-03 |
| RPL23A | 3.26 | 4.03E-03 |
| RPS5 | 3.28 | 4.41E-03 |
| RPS26 | 3.34 | 3.15E-03 |
| *RPL39; RPL39P5 | 3.63 | 1.67E-10 |
| ETF1 | 3.66 | 1.81E-12 |
| DPM1 | 3.66 | 2.17E-12 |

*Also changed in L3 vs normal skin

^Also changed in L2 vs normal skin
